# Effects of permissive hypercapnia on pulmonary and neurodevelopmental sequelae in extremely low birth weight infants: a meta-analysis

**DOI:** 10.1186/s40064-016-2437-5

**Published:** 2016-06-17

**Authors:** Jianglin Ma, Hui Ye

**Affiliations:** Department of Pediatrics, The First Affiliated Hospital, College of Medicine, Zhejiang University, Hangzhou, 310000 People’s Republic of China; Department of Pediatrics, Taizhou First People’s Hospital, Taizhou, 318020 People’s Republic of China

**Keywords:** Permissive hypercapnia, Lung protection, Neurodevelopmental sequelae, Extremely low birth weight infants, Meta-analysis

## Abstract

**Objectives:**

To perform a systematic review and meta-analysis of the efficacy and safety of permissive hypercapnia in extremely low birth weight infants.

**Methods:**

A systematic search of MEDLINE, EMBASE, the Cochrane Database of randomized trials. Eligibility and quality of trials were assessed, and data on study design, patient characteristics, and relevant outcomes were extracted.

**Results:**

Four studies that enrolled a total of 693 participants were selected. Meta-analysis revealed no effect of permissive hypercapnia on decreasing rates of bronchopulmonary dysplasia (BPD). Permissive hypercapnia also had no significant effect on mortality, intraventricular haemorrhage (IVH), IVH (grade 3–4), periventricular leukomalacia (PVL), necrotising enterocolitis (NEC), retinopathy of prematurity (ROP) or air leaks in extremely low birth weight infants. Neurodevelopmental outcomes were comparable at 18–22 months’ corrected age in two studies. permissive hypercapnia did not increase the risk of cerebral palsy, Mental Developmental Index <70, Psychomotor Developmental Index <70, visual deficit, or hearing deficit.

**Conclusions:**

Permissive hypercapnia did not reduce the rate of BPD in extremely low birth weight infants. The rates of mortality, IVH, PVL, NEC, ROP and neurodevelopmental outcomes did not differ between these two groups. These results suggest that permissive hypercapnia does not bring extra benefits in extremely low birth weight infants.

## Background

Advances in perinatal care has improved the survival rate of extremely low birth weight infants (Fanaroff et al. 2007), while most of them require mechanical ventilation in the first days of life. The neonatal mechanical ventilator could produce lung injury and increase long-term respiratory morbidity such as bronchopulmonary dysplasia (BPD) (Avery et al. 1987; Ramanathan and Sadesai 2008). Also, it could increase the risk of premature brain injury, such as periventricular leukomalacia (PVL) and intraventricular hemorrhage (IVH) (Ambalavanan and Carlo 2001; Okumura et al. 2001; Fabres et al. 2007).

Permissive hypercapnia is a ventilatory strategy that permits relatively high levels of partial pressure of arterial carbon dioxide (PaCO_2_) and always used to avoid lung injury and BPD (Miller and Carlo 2007). There are some evidence from experimental researches and clinical trials to support it. For example, in two animal models, therapeutic hypercapnia were demonstrated that it could reduce lung injury indices (Sinclair et al. 2002; Laffey et al. 2004). Rai and Ryu both have demonstrated that hypercapnia could alter lung structure and attenuate hyperoxia-induced fibrosis in newborn mice (Rai et al. 2004; Ryu et al. 2007). Two retrospective studies have reported that permissive hypercapnia might reduce the incidence of BPD (Kraybill et al. 1989; Garland et al. 1995). Recently, a large randomized controlled study was conducted in 359 extremely low birth weight infants to test whether permissive hypercapnia could reduce injury to the developing lung or bring other benefits, but the results noted that permissive hypercapnia did not reduce the rate of BPD or death in ventilated preterm infants (Thome et al. 2015).

However, permissive hypercapnia is still used in many ventilated preterm infants around the world. Whether permissive hypercapnia is still fit for using as an alternative to traditional ventilator support strategies should be elucidated. So a systematic review with a meta-analysis would be needed to establish whether permissive hypercapnia could reduce the incidence of BPD or bring other benefits or whether additional studies are needed.

## Methods

### Literature search

By using the search strategy of the Cochrane Neonatal Review Group, all researches were identified through electronic searches on Pubmed (from 1966 onward), Embase (from 1974 onward) and Cochrane Library with the terms “permissive”, “hypercapnia”, “infants” and “mechanical ventilation”. Only studies published in English were included.

### Inclusion and exclusion criteria

To be included in the meta-analyses, the studies needed to meet the following criteria. (1) the study was a randomized controlled trial; (2) each infant need mechanical ventilation; (3) the intervention was the elective use of permissive hypercapnia or conventional ventilation; (4) full text available in English. Studies were excluded according to the following exclusion criteria: (1) letters, editorials, expert opinions, case reports and reviews; (2) studies without usable data; (3) duplicate publications.

### Quality assessment

Two researchers evaluated the full text of the relevant studies and assessed the methodological quality according to the following criteria: allocation concealment, blinding of intervention, completeness of follow-up monitoring and blinding of outcome measurements.

### Outcome measures

For each study, the following data and outcome parameters were extracted independently by two reviewers: birth weight, gestational age, number of patients randomly assigned, mortality (death before 36 weeks postmenstrual age), BPD, IVH, PVL, necrotising enterocolitis (NEC), retinopathy of prematurity (ROP), air leaks, and long-term neurodevelopmental sequelae, including cerebral palsy, cognitive (Mental Developmental Index, MDI < 70) delay, psychomotor (Psychomotor Developmental Index, PDI < 70) delay, hearing and visual impairment. Hearing impairment was defined as the use of hearing aids. Vision impairment was defined as use of corrective or contact lenses, blind with some functional vision or no useful vision.

### Statistical analysis

All analysis of the extracted data were performed using Revman 5.1 software. Treatment outcomes and neurodevelopmental sequela for the dichotomous outcomes were expressed as relative risks (RRs) with 95 % confidence intervals (CIs) and numbers needed to treat. If heterogeneity was noted, then a random-effects model was used for the meta-analysis, otherwise a fixed-effects model was used.

## Results

### Study characteristics

A total of nine articles were identified by using the aforementioned search strategy. One prospective cohort study reported by Erika W. Hagen was excluded. The other eight studies were carefully read and four of them were excluded, because we could not get usable data. At last, four studies met the inclusion criteria for this article, randomly assigning a total of 693 infants. The overall quality of these 4 studies was fair to good (Table 1). As shown in Table 2, all these four studies included preterm infants with comparable gestational age and birth weight. Mechanical ventilation was used in the early period in all trials. All mechanically ventilated infants were randomly allocated to two different target ranges of PaCO_2_. But the rage of PaCO_2_ in permissive hypercapnia group was slightly different. In two studies, the permissive hypercapnia target range was 55–65 mmHg, the ranges of the other two were 45–55 and >52 mmHg.

**Table 1:** Methods of included studies

	Randomization	Concealment of allocation	Blinding of intervention	Completeness of follow-up monitoring	Blinding of assessment
Mariani et al. (1999)	Yes	Adequate	Yes	Yes	Yes
Carlo et al. (2002)	Yes	Adequate	Yes	Yes	Yes
Thome et al. (2006)	Yes	Adequate	Yes	Yes	Yes
Thome et al. (2015)	Yes	Adequate	Yes	Yes	Yes

**Table 2:** Patient characteristics in included studies

Group	No. of patients	PaCO_2_ (mmHg)	Birth weight, mean ± SD or Median (25th–75th percentile), g	Gestational age, mean ± SD or Median (25th–75th percentile) weeks
Mariani et al. (1999)				
PHC	24	45–55	852 ± 156	26 ± 1.0
Control	25	35–45	856 ± 173	26 ± 2.0
Carlo et al. (2002)				
PHC	109	>52	742 ± 130	25 ± 2.0
Control	111	<48	728 ± 135	25 ± 2.0
Thome et al. (2006)				
PHC	33	55–65	660 (353–944)	24.7 (23.0–28.9)
Control	32	35–45	621 (432–1204)	24.7 (23.0–28.3)
Thome et al. (2015)				
PHC	179	55–65	713 ± 156	25.6 ± 1.4
Control	180	40–50	709 ± 153	25.7 ± 1.3

*PHC* permissive hypercapnia

### Outcome parameters

**Fig. 1:**
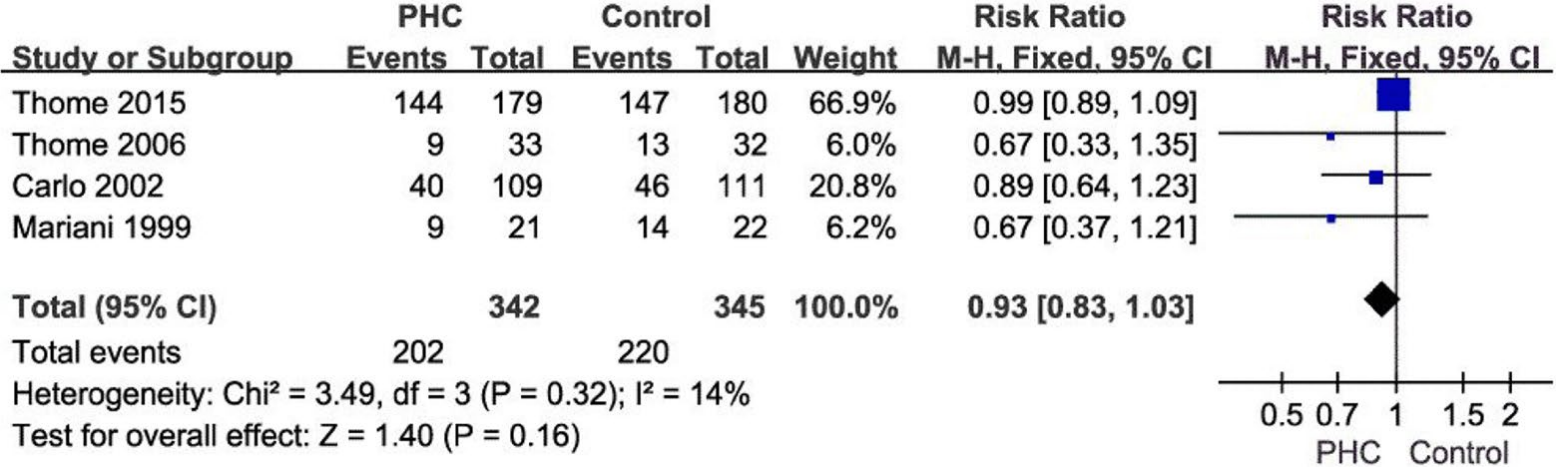
Forest plot of the BPD in extremely low birth weight infants who received permissive hypercapnia to controls. *CI* confidence interval, *df* degrees of freedom, *PHC* permissive hypercapnia, *M–H* Mantel–Haenszel

Data for the primary outcome were reported in all these four studies. There was no significant reduction in the risk of BPD in infants of PHC group compared to control group (four studies, 693 participants, over effect Z = 1.40 [*P* = 0.16]; pooled RR = 0.93; test of heterogeneity p = 0.32 and I^2^ = 14 %) (Fig. 1).

### Other effectiveness outcomes

**Fig. 2:**
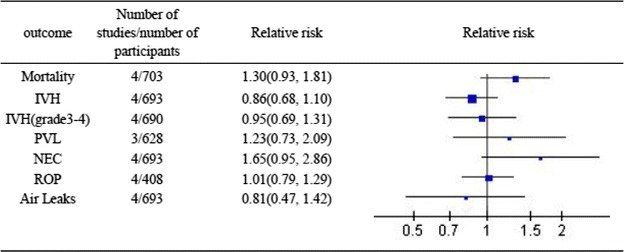
Overall results showing odds ratios and 95 % confidence intervals, calculated according to either fixed or random effects models, for the analysed other effectiveness parameters. Other effectiveness outcomes in extremely low birth weight infants who received permissive hypercapnia compared to controls. *IVH* intraventricular haemorrhag, *PVL* periventricular leukomalacia, *NEC* necrotising enterocolitis, *ROP* retinopathy of prematurity

Overall results were shown in Fig. 2. Permissive hypercapnia had no significant effect on mortality, IVH, IVH (grade 3–4), PVL, NEC, ROP and air leaks compared to controls (Fig. 2).

### Neurodevelopmental sequelae

**Fig. 3:**
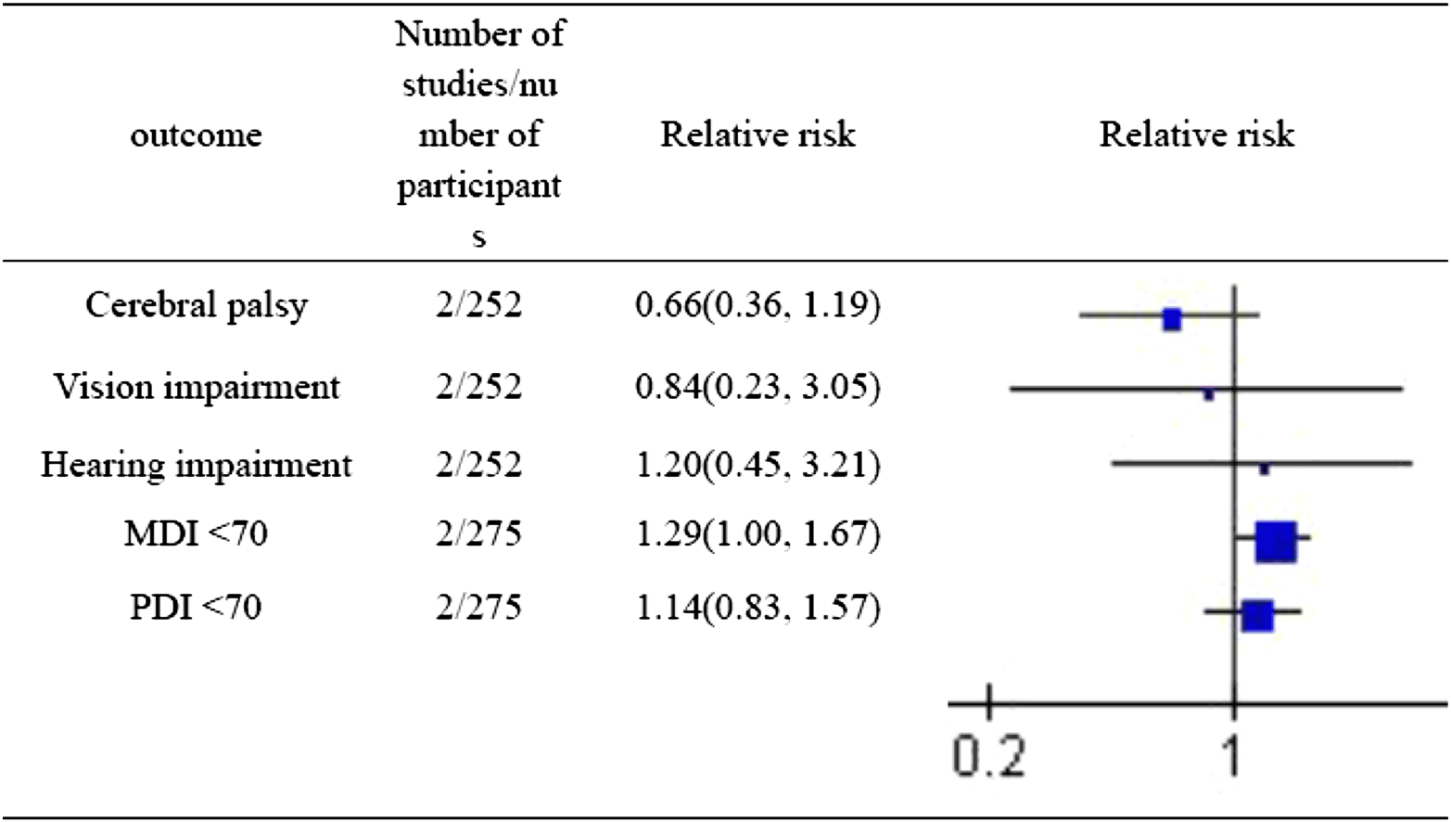
Overall results showing odds ratios and 95 % confidence intervals, calculated according to either fixed or random effects models, for the analysed neurodevelopmental Sequelae parameters. Neurodevelopmental Sequelae in extremely low birth weight infants who received permissive hypercapnia compared to controls. *MDI* Mental Developmental Index, *PDI* Psychomotor Developmental Index

Overall results were shown in Fig. 3. There was no difference in the risk of cerebral palsy, MDI < 70, PDI < 70, visual deficit, hearing deficit between infants who received PHC and controls (Fig. 3).

## Discussion

In this meta-analysis, we found that permissive hypercapnia did not reduce the rate of BPD in ventilated extremely low birth weight infants. The rates of mortality, IVH, IVH (grade 3–4), PVL, NEC, ROP and air leaks did not decrease in permissive hypercapnia group. There was no difference in the risk of cerebral palsy, MDI < 70, PDI < 70, visual deficit and hearing deficit between the PHC and control group.

Ventilatory support has reduced mortality of extremely low birth weight infants while resulted in an increase of the rates of BPD. BPD infants have many pulmonary complications and long-term respiratory consequences (Katz-Salomon et al. 2000; Doyle et al. 2006). In order to reduce the rate of BPD in ventilated preterm infants, one new ventilator strategy has been proposed and called as permissive hypercapnia. This strategy permits higher carbon dioxide tension (PaCO_2_) to reduce the risk of lung injury. In recent years, a lot of experimental and clinical studies have been conducted to determine if permissive hypercapnia is effective and safe in preterm infants (Mariani et al. 1999; Carlo et al. 2002; Thome et al. 2006; Morley et al. 2008; Finer et al. 2010; Dunn et al. 2011; Tapia et al. 2012). However, these trials that compare permissive hypercapnia with conventional ventilation have shown conflicting results. That is why we perform this study. Here, our meta-regression analysis showed the using of permissive hypercapnia in ventilatory support could not reduce the rate of BPD in extremely low birth weight infants over the years.

It is known that both extremes of PaCO_2_ could adversely affect the premature brain. Hypocapnia could increase the risk of periventricular leukomalacia (PVL) by impairing cerebral blood flow (Fujimoto et al. 1994; Wiswell et al. 1996). While severe hypercapnia is associated with an increased risk of intraventricular hemorrhage (Kaiser et al. 2006). However, the effect of mild hypercapnia on premature brain during permissive hypercapnic ventilation in extremely low birth weight infants is not clear. In this study, we found that the rates of IVH, IVH (grade 3–4), PVL did not differ in permissive hypercapnia group and control group. Besides, we found that permissive hypercapnia did not increase the rates of mortality, NEC, ROP or air leaks.

Some previous studies have found that both extreme fluctuations in PaCO_2_ and higher max PaCO_2_ were associated with worse neurodevelopmental outcomes (McKee et al. 2009). While we never know if permissive hypercapnia was associated with neurodevelopmental impairment. In this meta-analysis, we found that permissive hypercapnia strategy could not increase the risk of cerebral palsy, MDI < 70, PDI < 70, visual deficit or hearing deficit in extremely low birth weight infants.

This meta-analysis has two limitations. Firstly, only four studies engaged in and the sample size of this analysis was not big enough. So it may not detect small but clinically relevant differences in some important outcome parameters. Secondly, publication bias may occur as in other meta-analyses.

## References

[CR1] Ambalavanan N, Carlo WA (2001). Hypocapnia and hypercapnia in respiratory management of newborn infants. Clin Perinatol.

[CR2] Avery ME, Tooley WH, Keller JB, Hurd SS, Bryan MH, Cotton RB (1987). Is chronic lung disease in low birth weight infants preventable? A survey of eight centers. Pediatrics.

[CR3] Carlo WA, Stark AR, Wright LL, Tyson JE, Papile LA, Shankaran S (2002). Minimal ventilation to prevent bronchopulmonary dysplasia in extremely-low-birth-weight infants. J Pediatr.

[CR4] Doyle LW, Faber B, Callanan C (2006). Bronchopulmonary dysplasia in very low birth weight subjects and lung function in late adolescence. Pediatrics.

[CR5] Dunn MS, Kaempf J, de Klerk A, de Klerk R, Reilly M, Howard D (2011). Randomized trial comparing 3 approaches to the initial respiratory management of preterm neonates. Pediatrics.

[CR6] Fabres J, Carlo WA, Phillips V, Howard G, Ambalavanan N (2007). Both extremes of arterial carbon dioxide pressure and the magnitude of fluctuations in arterial carbon dioxide pressure are associated with severe intraventricular hemorrhage in preterm infants. Pediatrics.

[CR7] Fanaroff AA, Stoll BJ, Wright LL, Carlo WA, Ehrenkranz RA, Stark AR (2007). Trends in neonatal morbidity and mortality for very low birth weight infants. Am J Obstet Gynecol.

[CR8] Finer NN, Carlo WA, Walsh MC, Rich W, Gantz MG, Laptook AR (2010). Early CPAP versus surfactant in extremely preterm infants. N Engl J Med.

[CR9] Fujimoto S, Togari H, Yamaguchi N, Mizutani F, Suzuki S, Sobajima H (1994). Hypocarbia and cystic periventricular leukomalacia in premature infants. Arch Dis Child.

[CR10] Garland JS, Buck RK, Allred EN (1995). Hypocarbia before surfactant therapy appears to increase bronchopulmonary dysplasia risk in infants with respiratory distress syndrome. Arch Pediatr Adolesc Med.

[CR11] Kaiser JR, Gauss CH, Pont MM, Williams DK (2006). Hypercapnia during the first 3 days of life is associated with severe intraventricular hemorrhage in very low birth weight infants. J Perinatol.

[CR12] Katz-Salomon M, Gerner EM, Jonsson B (2000). Early motor and mental development in very preterm infants with chronic lung disease. Arch Dis Child Fetal Neonatal Ed.

[CR13] Kraybill EN, Runyan DK, Bose CL (1989). Risk factors for chronic lung disease in infants with birth weights of 751 to 1000 grams. J Pediatr.

[CR14] Laffey JG, Honan D, Hopkins N, Hyvelin JM, Boylan JF, McLoughlin P (2004). Hypercapnic acidosis attenuates endotoxin induced acute lung injury. Am J Respir Crit Care Med.

[CR15] Mariani G, Cifuentes J, Carlo W (1999). Randomized trial of permissive hypercapnia in preterm infants. Pediatrics.

[CR16] McKee LA, Fabres J, Howard G (2009). PaCO_2_ and neurodevelopment in extremely low birth weight infants. J Pediatr.

[CR17] Miller J, Carlo W (2007). Safety and effectiveness of permissive hypercapnia in the preterm infant. Curr Opin Pediatr.

[CR18] Morley CJ, Davis PG, Doyle LW, Brion LP, Hascoet JM, Carlin JB (2008). Nasal CPAP or intubation at birth for very preterm infants. N Engl J Med.

[CR19] Okumura A, Hayakawa F, Kato T, Itomi K, Maruyama K, Ishihara N (2001). Hypocarbia in preterm infants with periventricular leukomalacia: the relation between hypocarbia and mechanical ventilation. Pediatrics.

[CR20] Rai S, Engelberts D, Laffey JG, Frevert C, Kajikawa O, Martin TR (2004). Therapeutic hypercapnia is not protective in the in vivo surfactant-depleted rabbit lung. Pediatr Res.

[CR21] Ramanathan R, Sadesai S (2008). Lung protective ventilatory strategies in very low birth weight infants. J Perinatol.

[CR22] Ryu J, Sukkarieh M, Heldt G (2007) Effect of chronic hypercapnia on lung development. In: Abstracts of the American Thoracic Society (ATS), San Francisco, p A87

[CR23] Sinclair SE, Kregenow DA, Lamm WJ, Starr IR, Chi EY, Hlastala MP (2002). Hypercapnic acidosis is protective in an in vivo model of ventilator-induced lung injury. Am J Respir Crit Care Med.

[CR24] Tapia JL, Urzua S, Bancalari A, Meritano J, Torres G, Fabres J (2012). Randomized trial of early bubble continuous positive airway pressure for very low birth weight infants. J Pediatr.

[CR25] Thome UH, Carroll W, Wu TJ, Johnson RB, Roane C, Young D (2006). Outcome of extremely preterm infants randomized at birth to different PaCO_2_ targets during the first seven days of life. Biol Neonate.

[CR26] Thome UH, Genzel-Boroviczeny O, Bohnhorst B, Schmid M, Fuchs H, Rohde O (2015). Permissive hypercapnia in extremely low birthweight infants (PHELBI): a randomised controlled multicentre trial. Lancet Respir Med.

[CR27] Wiswell TE, Graziani LJ, Kornhauser MS, Stanley C, Merton DA, McKee L (1996). Effects of hypocarbia on the development of cystic periventricular leukomalacia in premature infants treated with high-frequency jet ventilation. Pediatrics.

